# A randomised controlled trial to investigate the effectiveness of sustained photoprotective behaviour in xeroderma pigmentosum after intervention

**DOI:** 10.1192/bjo.2021.764

**Published:** 2021-06-18

**Authors:** Tamara Searle, Jessica Walburn, Sam Norton

**Affiliations:** 1 IoPNN King's College London, University of Birmingham; 2 IoPPN King's College London

## Abstract

**Aims:**

This study aimed to investigate whether an intervention designed to improve photoprotective behaviours is effective at changing behaviour and whether any change could be maintained.

**Background:**

Xeroderma Pigmentosum (XP) is a rare condition in which patients are at risk of malignancies when exposed to ultra-violet radiation (UVR). Sufferers must take extra precautions to protect themselves from UVR. They must apply sunscreen to exposed skin, wear thick clothing, gloves, and a UVR-protective visor. Treatments include preventative photoprotective measures; the use of sunscreen and protective clothing. Additionally, frequent eye and skin examinations are required and swift removal of any premalignant lesions.

**Method:**

In this randomised controlled trial, 16 participants with XP were given questionnaires at 4 time points; baseline, post-intervention, 5 months and 9 months post-intervention. The intervention involved 7 one-on-one counselling sessions, as well as telephone consultations. Counselling sessions encouraged photoprotection adherence, self-efficacy and discussions of any barriers to improving photoprotective behaviour. This study focused on psychosocial variables, attitudes and photoprotection. Questionnaires included the photoprotection self-efficacy questionnaire, Self-Reported Behavioural Automaticity Index, Short Warwick-Edinburgh Mental Wellbeing Scale, Quality of Life and Brief Photoprotection Adherence Questionnaire.

**Result:**

The intervention was shown to have no significant effect on participants’ questionnaires scores. Univariate ANCOVA revealed a group effect between follow-up 1 (FU1) and follow-up 2 (FU2); η2 = 0.422 for self-efficacy in wearing photoprotective clothing. A group effect was identified from BL to FU1 and FU1 to FU2; η2 = 0.343 and η2 = 0.378 respectively in how often participants reapplied sunscreen to their face when outside for longer periods. Univariate ANCOVA revealed no group or time effect for the other outcome variables; for example, sunscreen self-efficacy.

**Conclusion:**

The intervention had no significant effect on photoprotective behaviour questionnaire scores. Future research could focus on recruiting more participants globally to generate more statistically powered results. Research should focus on producing a maintainable intervention so that any positive change would produce better long-term health outcomes. This study lays the foundations for future XP research, which will be vital to improve understanding and enhance photo protective behaviour.

